# Schisandrin C enhances type I IFN response activation to reduce tumor growth and sensitize chemotherapy through antitumor immunity

**DOI:** 10.3389/fphar.2024.1369563

**Published:** 2024-08-07

**Authors:** Huijie Yang, Xiaoyan Zhan, Jia Zhao, Wei Shi, Tingting Liu, Ziying Wei, Hui Li, Xiaorong Hou, Wenqing Mu, Yuanyuan Chen, Congyang Zheng, Zhongxia Wang, Shengli Wei, Xiaohe Xiao, Zhaofang Bai

**Affiliations:** ^1^ School of Chinese Materia Medica, Beijing University of Chinese Medicine, Beijing, China; ^2^ China Military Institute of Chinese Materia, The Fifth Medical Center of PLA General Hospital, Beijing, China; ^3^ Senior Department of Hepatology, The Fifth Medical Center of PLA General Hospital, Beijing, China; ^4^ National Key Laboratory of Kidney Diseases, Beijing, China; ^5^ School of Pharmacy, North Sichuan Medical College, Nanchong, China; ^6^ School of Life Sciences, Beijing University of Chinese Medicine, Beijing, China; ^7^ The Third Affiliated Hospital of Zunyi Medical University (The First People’s Hospital of Zunyi), Zunyi, China; ^8^ Department of Nutrition, The Fifth Medical Center of PLA General Hospital, Beijing, China

**Keywords:** Schisandrin C, cGAS-STING pathway, antitumor immunity, type I interferon, CD8^+^ T cell, NK cell

## Abstract

With the advancing comprehension of immunology, an increasing number of immunotherapies are being explored and implemented in the field of cancer treatment. The cGAS-STING pathway, a crucial element of the innate immune response, has been identified as pivotal in cancer immunotherapy. We evaluated the antitumor effects of *Schisandra chinensis* lignan component Schisandrin C (SC) in 4T1 and MC38 tumor-bearing mice, and studied the enhancing effects of SC on the cGAS-STING pathway and antitumor immunity through RNA sequencing, qRT-PCR, and flow cytometry. Our findings revealed that SC significantly inhibited tumor growth in models of both breast and colon cancer. This suppression of tumor growth was attributed to the activation of type I IFN response and the augmented presence of T cells and NK cells within the tumor. Additionally, SC markedly promoted the cGAS-STING pathway activation induced by cisplatin. In comparison to cisplatin monotherapy, the combined treatment of SC and cisplatin exhibited a greater inhibitory effect on tumor growth. The amplified chemotherapeutic efficacy was associated with an enhanced type I IFN response and strengthened antitumor immunity. SC was shown to reduce tumor growth and increase chemotherapy sensitivity by enhancing the type I IFN response activation and boosting antitumor immunity, which enriched the research into the antitumor immunity of *S. chinensis* and laid a theoretical basis for its application in combating breast and colon cancer.

## 1 Introduction

With the advancing comprehension of immunology, an increasing number of immunotherapies are being explored and implemented in the field of cancer treatment (Zhao et al., 2021). The cyclic GMP-AMP synthase (cGAS)-stimulator of interferon genes (STING) pathway, a vital component of the innate immune system, has emerged as pivotal in cancer immunotherapy (Kwon and Bakhoum, 2020). Tumor-derived DNA activates the cGAS-STING pathway, instigating type I interferon (IFN) production. In contrast to regular cells, tumor cells exhibit characteristics such as genetic instability and heightened oxidative stress (Luo et al., 2009; Roers et al., 2016; Mackenzie et al., 2017; Bakhoum and Cantley, 2018; Bakhoum et al., 2018). These factors contribute to the fragility of their nuclear and mitochondrial DNA, making them susceptible to leakage, often observable through the presence of micronuclei and chromatin debris (Woo et al., 2014; Chen et al., 2017; Mackenzie et al., 2017). cGAS, a cellular DNA sensor, plays a crucial role in dsDNA recognition (Ablasser et al., 2013; Cai et al., 2014; Härtlova et al., 2015; Harding et al., 2017; Ablasser and Chen, 2019), activating STING and subsequently IFN regulatory factor 3 (IRF3), upregulating interferon-stimulated genes (ISGs) type I IFN production (Ishikawa and Barber, 2008; Ishikawa and Barber, 2011; Chen et al., 2016).

The cGAS-STING pathway can modulate various stages of the cancer-immunity cycle (Anandappa et al., 2020; Lv et al., 2020; Du et al., 2022; Pan et al., 2023). A diverse array of chemokines, including CXCL9, CXCL10, and CCL5, which are triggered by type I IFN signaling, play integral roles in facilitating cytotoxic T lymphocytes (CTLs) trafficking and infiltration (Zheng et al., 2020). Moreover, type I IFN can activate CTLs and NK cells, thereby promoting their tumor-cell killing capabilities (Lv et al., 2020). Deficiencies in either STING or IRF3 hinder CD8^+^ T cells activation within tumors, underlining the significance of the cGAS-STING pathway in antitumor immunity (Zhu YY. et al., 2019). With growing acknowledgment of its importance, the cGAS-STING pathway is coming to the forefront as a critical focal point for anti-cancer therapies. An array of STING agonists has been developed, including cyclic dinucleotides, their derivatives, DMXAA, their analogues, and small molecule agonists (Zheng et al., 2020). Furthermore, existing research indicates that both radiotherapy and chemotherapy can activate the STING pathway (Flood et al., 2019). Thus, focusing on the cGAS-STING pathway presents a potential avenue for discovering new antitumor drugs. Additionally, combining STING pathway enhancers with other anticancer strategies may enhance antitumor efficacy (Hines et al., 2023).

Our previous studies have confirmed that Schisandrin C (SC), a lignan in *Schisandra chinensis* (Turcz.) Baill., enhances cGAS-STING pathway activation, thereby exerting an anti-HBV effect (Zhao et al., 2023). However, the antitumor effects and specific mechanisms of SC have not been extensively investigated and merit further exploration.

**FIGURE 1:**
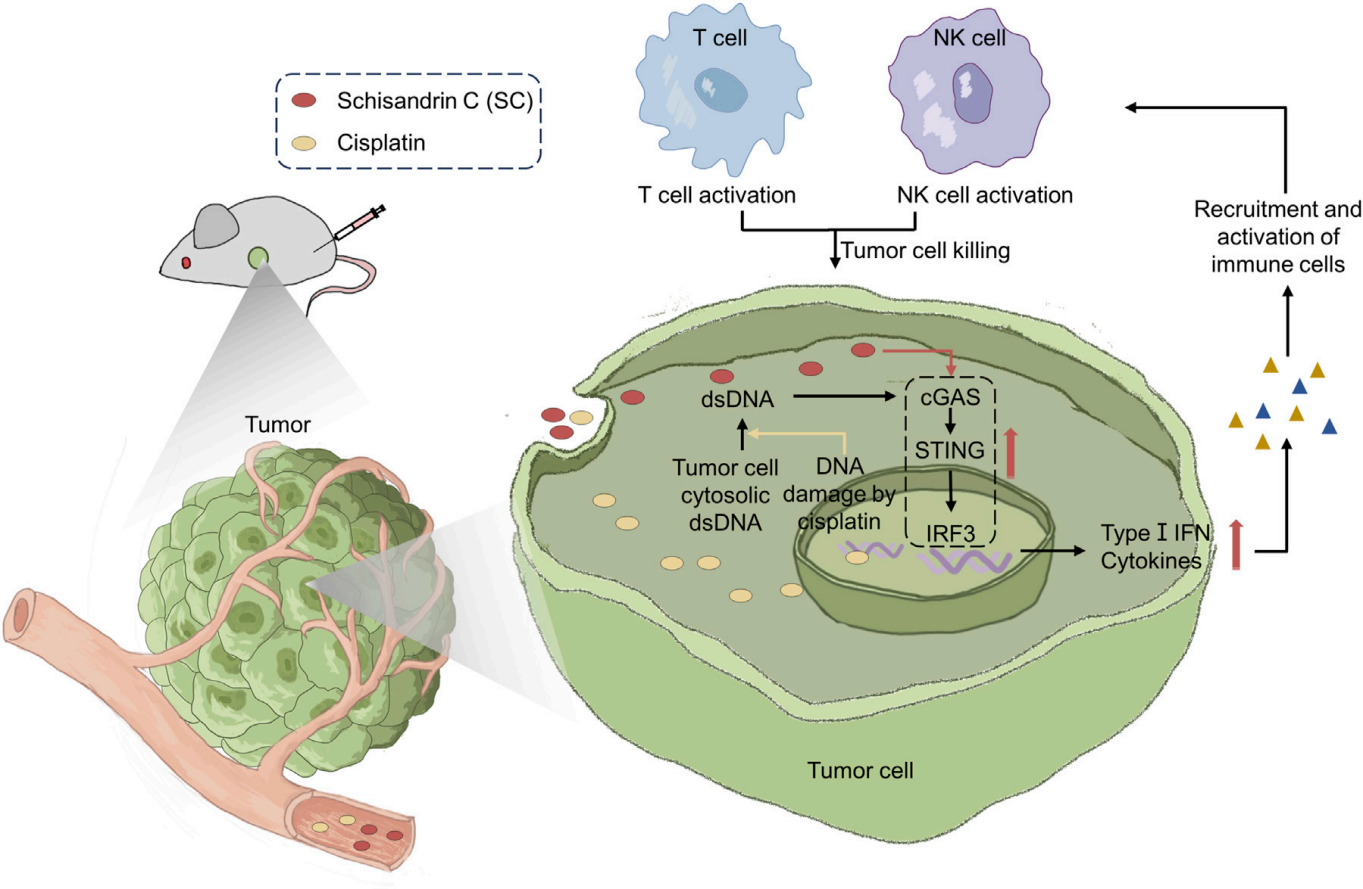
Schisandrin C enhances type I IFN response activation to reduce tumor growth and sensitize chemotherapy through antitumor immunity.

Our data demonstrated SC significantly inhibited tumor growth in both 4T1 breast cancer and MC38 colon cancer mouse models. The antitumor mechanism involves T cell and NK cell-mediated immune responses dependent on type I IFN. Consistently, we demonstrated that SC enhanced the cGAS-STING pathway activated by cisplatin, thereby activating the type I IFN response to promote antitumor immune responses and exert a synergistic antitumor effect (Figure 1 is a graphical summary of the article). Given these findings, SC not only enhances antitumor immunity but also shows promising potential as a candidate for cancer treatment and adjunctive chemotherapy.

## 2 Materials and methods

### 2.1 Materials

Schisandrin C (JOT-10307) was purchased from Chengdu Pufei De Biotech Co., Ltd. (Chengdu, China). Cisplatin (T1564) was purchased from TargetMol (United States). DMSO (HY-Y0320) was purchased from MedChemExpress (United States). Color Prestained Protein marker (20AB01) was purchased from GenStar (Beijing, China). DMEM Medium (CM10013) and penicillin-streptomycin were purchased from MacGene (Beijing, China). FBS was purchased from VivaCell (Shanghai, China). Mouse 1× Lymphocyte Separation Medium was purchased from Dakewe (Shenzhen, China). The antibodies used include: rabbit monoclonal anti-pIRF3 (GTX86691) was from GeneTex (USA). IRF3 polyclonal antibody (11312-1-AP), and TMEM173/STING polyclonal antibody (19851-1-AP) were from the Proteintech Group (USA). HSP90 antibody (sc-101494) was purchased from Santa Cruz (USA). RBC Lysis Buffer (420301), TruStain FcX™ PLUS (anti-mouse CD16/32) (S17011E, 156603), FITC anti–mouse CD45 (30-F11, 103107), Brilliant Violet 421™ anti-mouse CD3ε (145-2C11, 100335), PE/Cyanine7 anti-mouse CD4 (RM4-5, 100527), PE anti–mouse CD8a (53-6.7, 100708), Zombie Aqua™ Fixable Viability Kit (423101), APC anti-mouse NK1.1 (PK136, 108709) were purchased from BioLegend (United States).

### 2.2 Animals and syngeneic model

BALB/c and C57BL/6 mice were procured from SPF Biotechnology Co., Ltd. (Beijing, China). All experimental protocols involving animals were performed in strict adherence to the guidelines established by the Animal Care Committee of the Fifth Medical Center of PLA General Hospital.

Before tumor cell injection, the mice, aged between 5 to 6 weeks and closely age-matched, had one of their flanks shaved. Subsequently, tumor cells were administered into the shaved flank. Volume of the tumor was measured every 3 days and computed using the given formula: (length) × (width)^2^/2. Eventually, the mice were sacrificed on day 21.

### 2.3 Cell culture

The 4T1 cells were cultivated using the RPMI 1640 medium (MacGene). Meanwhile, DMEM medium was employed for growing the MC38 cells. The cells were incubated in a humidified atmosphere with 5% CO_2_ at 37°C.

### 2.4 Cell viability assay

MC38 cells and 4T1 cells were seeded in 96-well plates at a density of 1 × 10^5^ cells/mL, each well containing 100 μL. The following day, these cells underwent treatment with various SC concentrations over 24 h. Cell viability was subsequently assessed with the Cell Counting Kit-8, following the manufacturer’s strict procedures.

### 2.5 Quantitative real-time PCR (qRT-PCR)

Total RNA was isolated using Trizol (Sigma) and synthesized into cDNA using reverse transcription reagents (RT Master Mix for qPCR II, MedChemExpress). Subsequently, the cDNA was amplified through a Quant Studio 6 real-time PCR instrument employing SYBR Green qPCR Master Mix (MedChemExpress). Actin was used to normalize mRNA expression levels. The sequences for the mouse quantitative PCR primers are shown in Table 1.

**TABLE 1:** Quantitative PCR primer sequences.

Target gene	Sequence (5'-3')
Mouse actin	GGC​TGT​ATT​CCC​CTC​CAT​CG
	CCA​GTT​GGT​AAC​AAT​GCC​ATG​T
Mouse IFNβ	TCC​GAG​CAG​AGA​TCT​TCA​GGA​A
	TGCAAC CACCACTCATTCTGAG
Mouse CXCL 10	ATC​ATC​CCT​GCG​AGC​CTA​TCC​T
	GAC​CTT​TTT​TGG​CTA​AAC​GCT​TTC
Mouse ISG15	GGT​GTC​CGT​GAC​TAA​CTC​CAT
	CTG​TAC​CAC​TAG​CAT​CAC​TGT​G
Mouse IFIT1	GAA​CCC​ATT​GGG​GAT​GCA​CAA​CCT
	CTT​GTC​CAG​GTA​GAT​CTG​GGC​TTC​T

### 2.6 Analysis of TILs and NK cells by flow cytometry

On the 21st day after the tumor cells were inoculated, the tumors were removed, weighed, mechanically minced, and incubated for 20 min in Collagenase IV (1 mg/mL) and DNase I (40 μg/mL) at 37°C. The cells were then filtered to prepare a single-cell suspension. We enriched lymphocytes using a mouse lymphocyte separation medium. The enriched cells were blocked with anti-CD16/32 antibodies and then stained with surface antibodies at room temperature for 25 min. The antibodies used are listed in the antibody section above. The BD FACSCanto flow cytometry was used to analyze stained cells.

### 2.7 RNA-Seq

Total RNA was extracted and subjected to quality testing, followed by the synthesis, purification, and identification of double-stranded cDNA using messenger RNA procedures. Subsequently, the construction and sequencing of sample libraries were performed. The high-throughput Illumina HiSeq 4000 sequencing platform was utilized to generate raw data. Sample processing and online sequencing were completed by the Sangon Biotech (Shanghai) Co., Ltd. DESeq2 was used to identify the differentially expressed genes (DEGs) (|log_2_FC| ≥ 1, and *p* ≤ 0.05) between each group. After constructing appropriate gene sets, gene set enrichment analysis (GSEA) analyses were conducted using the Cluster Profiler R package.

### 2.8 Western blot analysis

Cell lysates were prepared using a loading buffer that includes a radioimmunoprecipitation assay buffer. Following this, the sample mixtures were denatured at a temperature of 105°C for 15 min. Subsequently, equal protein quantities were subjected to 10% SDS-PAGE, followed by transfer onto 0.22 µm PVDF membranes. To minimize non-specific interactions, membranes were blocked with 5% non-fat milk at room temperature for 1 hour. Overnight incubation at 4°C with specific primary antibodies preceded a 1-h room temperature incubation with secondary antibodies. Signal detection was carried out with the enhanced chemiluminescent reagent, supplied by Promega (United States), and analyses were performed subsequently.

### 2.9 Statistics

Statistical analyses were conducted utilizing the GraphPad Prism 8.0 software. The scrutiny of differences among multiple groups was accomplished through a one-way ANOVA, whereas the assessment of disparities between two individual groups was executed employing an unpaired Student’s t-test. Survival curves were delineated using the Kaplan-Meier estimator, and the comparative analysis of survival rates was performed with the log-rank (Mantel-Cox) test. All experimental data were conveyed as the mean ± SD. *p* < 0.05 was considered significant.

## 3 Results

### 3.1 SC inhibits tumor growth in 4T1 and MC38 tumor-bearing mice

**FIGURE 2:**
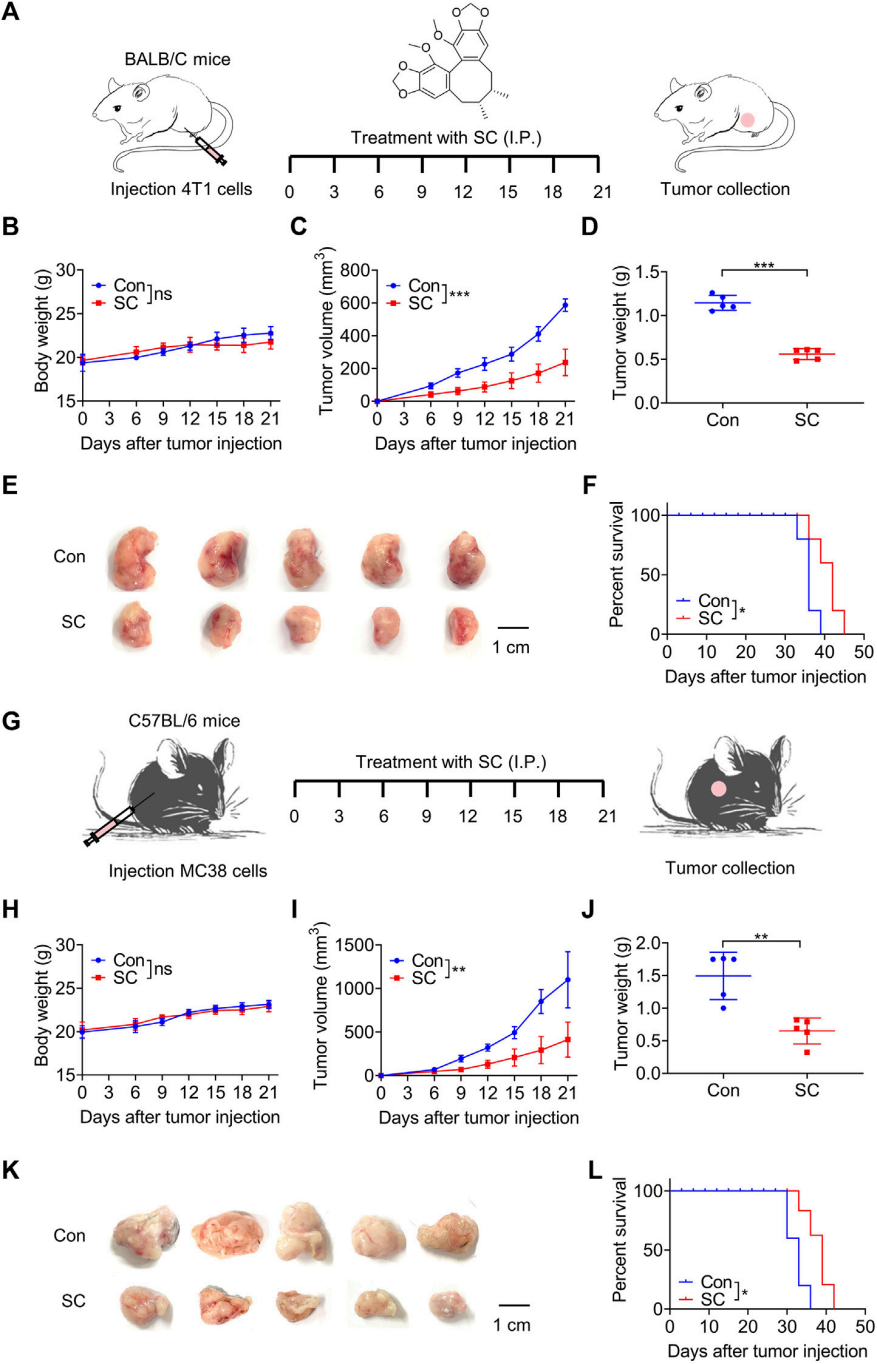
SC inhibits 4T1 and MC38 tumor growth. **(A)** Experiment design for BALB/c mice inoculated with approximately 1 × 10^5^ 4T1 cells. **(B)** Body weight changes during the whole experimental period. **(C)** Tumor volume changes during the whole experimental period. **(D)** Tumor weight. **(E)** Image of tumor mass. **(F)** Kaplan-Meier survival curves for BALB/c mice inoculated with 4T1 cells. **(G)** Experiment design for C57BL/6 mice inoculated with approximately 1 × 10^5^ MC38 cells. **(H)** Body weight changes during the whole experimental period. **(I)** Tumor volume changes during the whole experimental period. **(J)** Tumor weight. **(K)** Image of tumor mass. **(L)** Kaplan-Meier survival curves for C57BL/6 mice inoculated with MC38 cells. Data are shown as mean ± SD, ns, not significant; ^*^ *p* < 0.05, ^**^ *p* < 0.01, and^***^ *p* < 0.001 (unpaired Student’s t-test).

We first investigated the antitumor effect of SC in 4T1 tumor-bearing mice (Figure 2A). There was no significant difference in body weight between the groups (Figure 2B), suggesting that SC had no severe side effects on mice. Importantly, our results showed that the SC-treated group experienced a significant delay in tumor growth (Figures 2C–E). Tumors in the SC treatment group (30 mg/kg) demonstrated a significant volume reduction compared to the control group after 21 days of treatment. Concurrently, the tumor weight in the SC treatment group significantly decreased. Consistently, photographs of the tumors taken on day 21 clearly illustrated the retardation of tumor growth. To further investigate the therapeutic impact of SC, we conducted a survival analysis. The overall survival was significantly improved in the SC-treated group compared with the vehicle group (Figure 2F). Therefore, our results in the 4T1 murine tumor model strongly suggested that SC could effectively inhibit tumor growth.

Subsequently, we established a mouse model using MC38 colon cancer cells to assess the potential universality of SC’s antitumor effect (Figure 2G). SC significantly inhibited tumor growth in comparison to the control group, without affecting body weight (Figures 2H, I). Tumors were significantly smaller in the SC-treated group compared with the control group (Figures 2J, K). Moreover, mice treated with SC exhibited a significantly higher survival rate (Figure 2L). Therefore, our findings strongly suggested that SC could effectively inhibit tumor growth from both 4T1 and MC38 murine tumor models, with no observed clinical signs of toxicity in mice.

### 3.2 SC reduces tumor growth by enhancing type I IFN response in a cGAS-STING pathway-dependent manner

**FIGURE 3:**
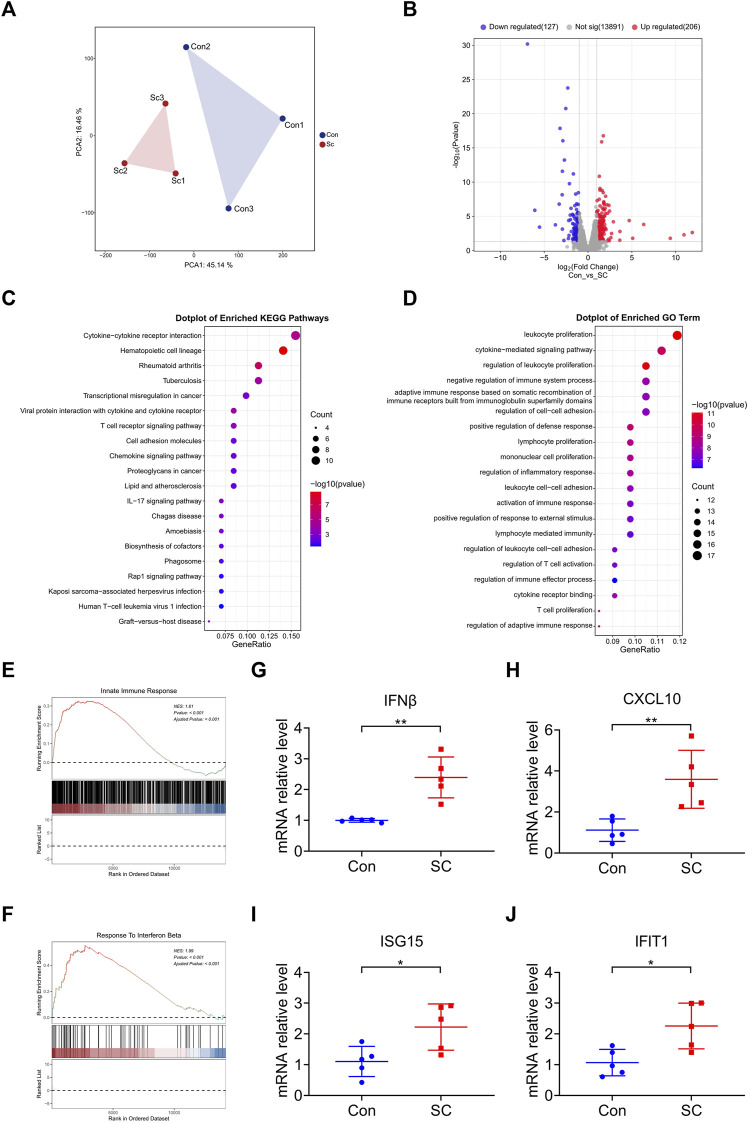
SC reduces tumor growth by enhancing type I IFN response in a cGAS-STING pathway-dependent manner. **(A)** PCA of RNA sequencing of tumors formed by MC38 cells left untreated or treated with SC in C57BL/6 mice. *n* = 3 tumors. **(B)** The expression levels of 333 genes were significantly altered, visualized using a volcano plot; 206 genes were upregulated and 127 genes were downregulated. **(C,D)** KEGG pathway analysis and GO pathway analysis showed upregulation pathways following treatment with SC compared to the untreated group. **(E,F)** GSEA revealed the indicated gene signatures. Notably, the gene signatures for the innate immune response **(E)** and response to IFNβ **(F)** were markedly enriched in the tumors following treatment with SC, particularly when compared to the untreated group. **(G–J)** The expressions of IFNβ **(G)**, CXCL10 **(H)**, ISG15 **(I)**, and IFIT1 **(J)** mRNA in tumors were detected by qRT-PCR. Data are shown as mean ± SD, ^*^ *p* < 0.05, ^**^ *p* < 0.01 (unpaired Student’s t-test).

To elucidate the mechanism through which SC mediates the delay in tumor progression, we conducted a comprehensive RNA sequencing analysis of the tumor tissues. Subsequent Principal Component Analysis (PCA) confirmed that the SC treatment significantly altered gene expression profiles (Figure 3A). In comparison with the control group, we identified 206 upregulated and 127 downregulated genes in SC-treated tumor tissues (Figure 3B). A Kyoto Encyclopedia of Genes and Genomes (KEGG) pathway analysis unveiled SC exerted antitumor effects by activating cytokine-cytokine receptor interaction, chemokine signaling pathway and T cell receptor signaling pathway (Figure 3C), affirming the critical role of antitumor immunity in SC’s inhibition of tumor growth. A Gene Ontology (GO) enrichment analysis correlated with KEGG findings, pointing toward immune-related biological processes as the major site of upregulated gene enrichment (Figure 3D).

Motivated by the outcomes of the KEGG and GO analyses, we supplemented these findings with a Gene Set Enrichment Analysis (GSEA) to gain further insights into immune function involvement. GSEA analysis revealed that the SC treatment resulted in a higher degree of innate immune response and response to IFNβ, highlighting the participation of type I IFN response in the antitumor effect (Figures 3E, F). It is widely recognized that type I IFN are the downstream cytokines regulated by the cGAS-STING signaling pathway (Zheng et al., 2020). Moreover, our previous studies have shown that SC activates the cGAS-STING pathway (Zhao et al., 2023), prompting us to focus further on this mechanism.

During tumor progression, the DNA of tumor cells is relatively fragile and prone to leakage, leading to activation of the cGAS-STING signaling pathway (Mackenzie et al., 2017). According to analysis, SC exerted subsequent antitumor effects by enhancing the activation of the cGAS-STING pathway. To verify the enrichment results, we analyzed the mRNA expression of IFNβ, CXCL10, ISG15, and IFIT1 within tumors. Consistently, SC markedly enhanced the transcription of these cytokines in tumors formed by MC38 cells (Figures 3G–J), testifying to type I IFN response’s critical role in SC’s antitumor mechanism. Therefore, the treatment of SC increased the activation of the cGAS-STING pathway, leading to the further expression of type I IFN and various inflammatory cytokines, sparking off an innate immune response.

### 3.3 SC boosts T cell response and NK cell response in tumors

**FIGURE 4:**
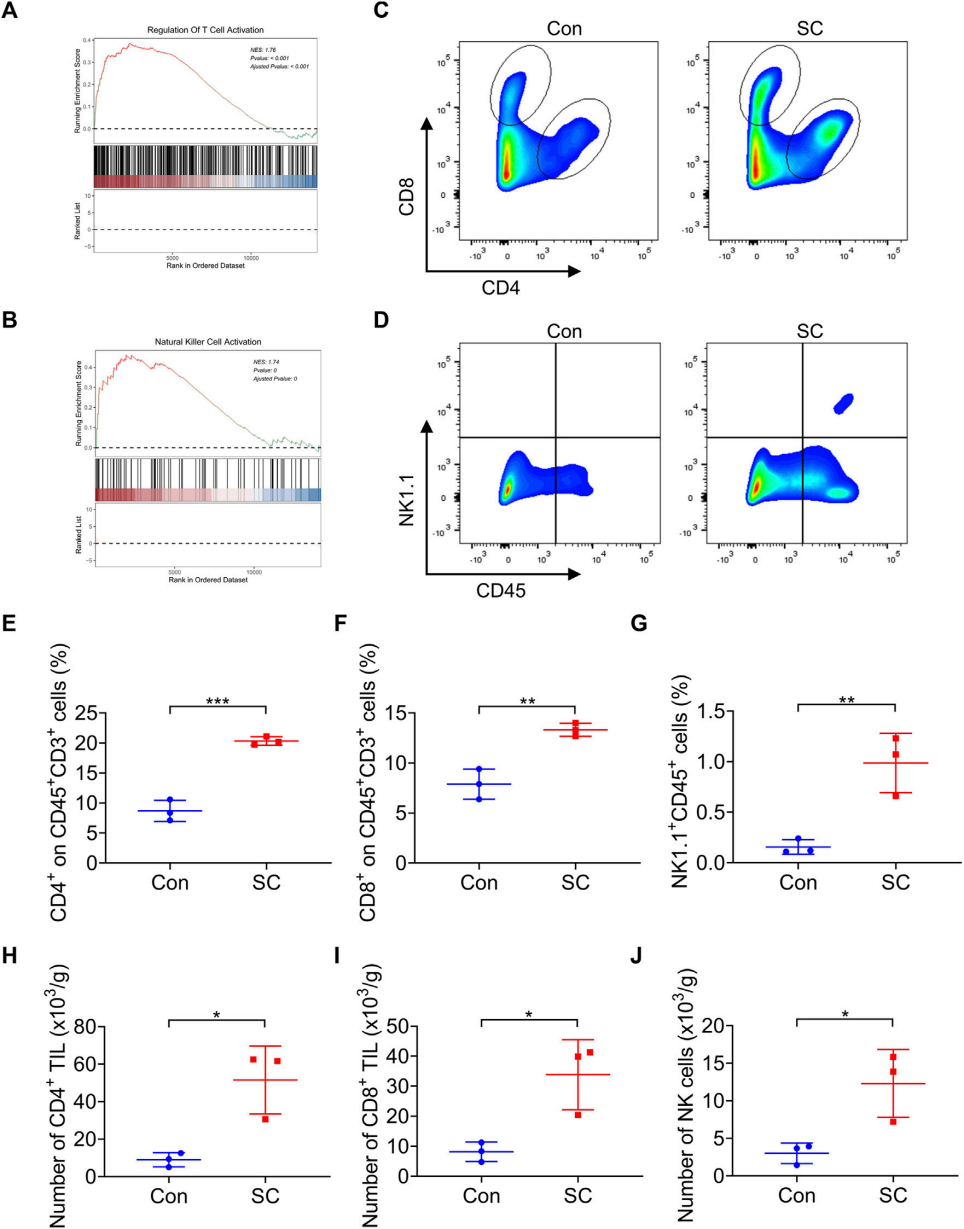
SC boosts T cell response and NK cell response in tumors. **(A,B)** GSEA showed that the activated T cell **(A)** and NK cell **(B)** signatures were enriched in the tumors after treatment with SC in comparison with the untreated group. **(C,D)** Representative flow cytometry data of CD4^+^ and CD8^+^ T cells or NK^+^ cells in tumors formed by MC38 cells left untreated or treated with SC. **(E–G)** Quantification of the results from **(C,D)**. **(H–J)** Average numbers of tumor-infiltrating CD4^+^ T cells **(H)**, CD8^+^ T cells **(I)**, and NK cells **(J)** of tissues from transplanted MC38 tumors grown in C57BL/6 mice left untreated or treated with SC. Data are shown as mean ± SD, ^*^ *p* < 0.05, ^**^ *p* < 0.01, and ^***^ *p* < 0.001 (unpaired Student’s t-test).

Evidence from prior research indicates that type I IFN catalyzes the upregulation of several chemokines essential for directing the infiltration of T cells and NK cells into tumor sites (Zheng et al., 2020). Intriguingly, Gene Set Enrichment Analysis (GSEA) disclosed that treatment with SC led to an enriched presence of activated T cells and NK cells (Figures 4A, B), which were pivotal in the elimination of tumor cells. To examine the involvement of T cells and NK cells in the antitumor process of SC, we analyzed the levels of tumor-infiltrating T cells and NK cells in both vector control and SC-treated tumors by flow cytometry (Figures 4C, D). Notably, SC-treated mice exhibited an elevated frequency of CD4^+^ T cells, CD8^+^ T cells, and NK cells within the tumors compared to control (Figures 4E–G). Correspondingly, a marked increase in the infiltration of both CD4^+^ and CD8^+^ T cells, alongside NK cells, was observed in tumors from the SC-treated group (Figures 4H–J). In conclusion, our data suggested that SC effectively inhibited tumor growth by initiating T cell and NK cell-driven immune responses in a type I IFN-dependent manner.

### 3.4 SC enhances cisplatin-induced type I IFN response via the cGAS-STING pathway in MC38 cells and 4T1 cells

Fascinatingly, recent studies propose that the amalgamation of cGAS-STING pathway enhancers with chemotherapy drugs potentially amplifies antitumor effects (Liu et al., 2023). In addition to the leaked DNA of tumor cells that are susceptible to genotoxic stress during tumor progression, chemotherapy with platinum drugs, such as cisplatin, can also cause DNA damage and activate the cGAS-STING signaling pathway (Grabosch et al., 2019; Glorieux et al., 2022). Therefore, to maximize the antitumor effect of SC, we attempted to verify whether SC could enhance the activity of the cisplatin-induced cGAS-STING pathway to induce type I IFN response in MC38 cells and 4T1 cells, thereby enhancing antitumor immunity against DNA damage caused by chemotherapy and further improving antitumor efficacy.

**FIGURE 5:**
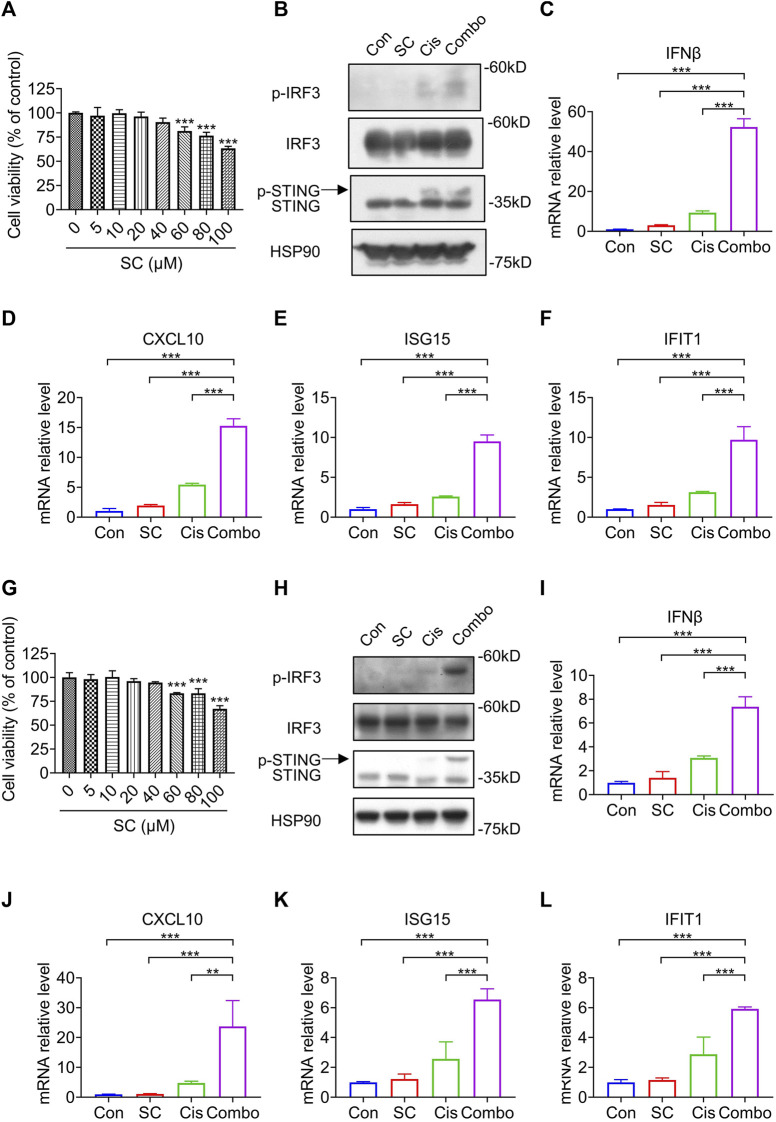
SC enhances cisplatin-induced type I IFN responses via the cGAS-STING pathway in MC38 cells and 4T1 cells. **(A)** Cell viability of MC38 cells treated with SC for 24 h. **(B)** Immunoblotting for indicated signaling molecules in MC38 cells, with or without cisplatin (30 μM) and SC (15 μM) treatment. **(C–F)** qRT-PCR analysis of IFNβ, CXCL10, ISG15, and IFIT1 transcripts in MC38 cells under the same conditions. **(G)** Cell viability of 4T1 cells treated with SC for 24 h. **(H)** Immunoblotting for indicated signaling molecules in 4T1 cells, with or without cisplatin (30 μM) and SC (15 μM) treatment. **(I–L)** qRT-PCR analysis of IFNβ, CXCL10, ISG15, and IFIT1 transcripts in 4T1 cells under similar conditions. Data are shown as mean ± SD, ^**^ *p* < 0.01, and ^***^ *p* < 0.001 (one-way ANOVA with Dunnett’s post-hoc test).

Before further exploring the regulatory role of SC in the cisplatin-induced cGAS-STING pathway, we initially assessed the cytotoxicity of SC in MC38 cells. SC was applied to MC38 cells for 24 h before assessing cell viability. The results showed that SC exhibited minimal toxicity (Figure 5A), consistent with our previous observations in normal cells (Zhao et al., 2023). Subsequently, we assessed the impact of SC on the cisplatin-induced cGAS-STING pathway in MC38 cells, utilizing a concentration of 15 μM based on previous studies (Zhao et al., 2023). Immunoblotting analyses revealed a notable increase in the protein levels of phosphorylated IRF3 and STING in the combination group of SC and cisplatin, as compared to using cisplatin alone (Figure 5B). In addition, we detected mRNA expression of several ISGs downstream of cGAS-STING in MC38 cells: IFNβ, CXCL10, ISG15, and IFIT1 (Figures 5C–F). Consistent with previous results, SC synergistically enhanced the transcription of these genes induced by cisplatin. Consistent with the results in MC38 cells, SC also enhanced the protein levels of phosphorylated IRF3 and STING as well as the transcription levels of downstream genes such as IFNβ, CXCL10, ISG15, and IFIT1 in 4T1 cells (Figures 5G–L). Collectively, SC boosted the cisplatin-induced type I IFN response in a cGAS-STING dependent way. In MC38 cells and 4T1 cells, SC alone didn't increase the expression of downstream factors related to the cGAS-STING signaling pathway, consistent with our previous findings that SC requires signaling stimulation to promote expression. Conversely, *in vivo* analyses revealed a distinctive outcome whereby the solitary treatment with SC managed to catalyze the activation of pathway-associated factors. This difference can be attributed to the fragility and proneness to leakage of tumor cell DNA during tumoral progression, thereby precipitating the activation of the cGAS-STING signaling pathway. SC enhances the activation of the cGAS-STING pathway, subsequently exerting its antitumor effects.

### 3.5 The enhancement of chemotherapy efficacy by SC is related to boosting the cisplatin-induced type I IFN response as well as enhancing antitumor immunity

**FIGURE 6:**
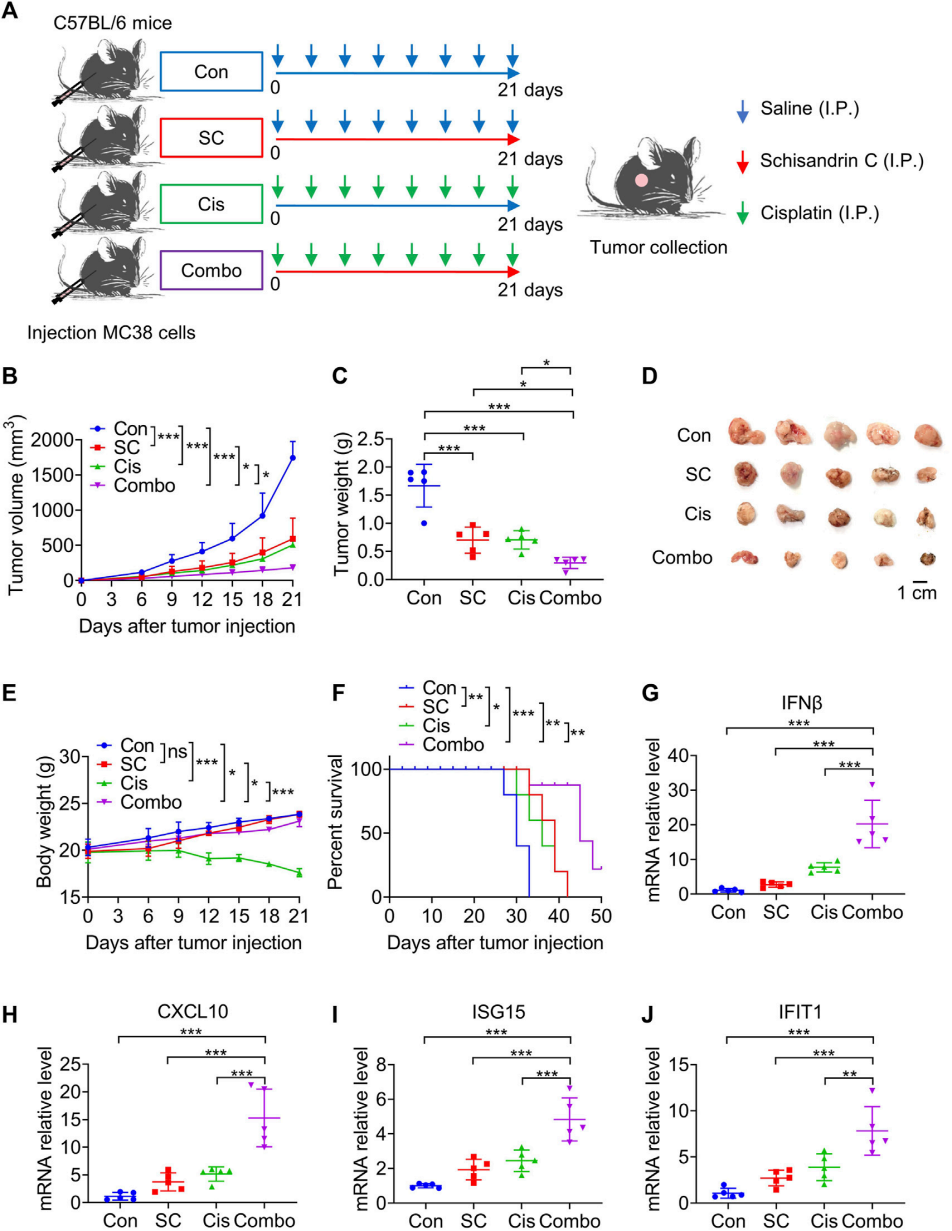
SC enhances the chemotherapeutic effect of cisplatin via the cGAS-STING pathway. **(A)** Experiment design for C57BL/6 mice inoculated with approximately 1 × 10^5^ MC38 cells. **(B)** Tumor volume changes during the whole experimental period. **(C)** Tumor weight. **(D)** Image of tumor mass. **(E)** Body weight changes during the whole experimental period. **(F)** Kaplan-Meier survival curves for C57BL/6 mice inoculated with MC38 cells. **(G–J)** Quantitative RT-PCR measurement of IFNβ, CXCL10, ISG15, and IFIT1 transcripts in MC38 tumor left unstimulated or stimulated with cisplatin (2 mg/kg) in the absence or presence of SC (30 mg/kg). Data are shown as mean ± SD, ^*^ *p* < 0.05, ^**^ *p* < 0.01, and^***^ *p* < 0.001 (one-way ANOVA with Dunnett’s post-hoc test).

Upon establishing that SC augmented the cisplatin-induced activation of the cGAS-STING pathway *in vitro*, our focus then shifted to examining whether SC could enhance the antitumor efficacy of cisplatin, specifically through the type I IFN response pathway. The efficacy of the antitumor action was evaluated in MC38 tumor-bearing mice that were either unstimulated or stimulated with cisplatin, under conditions both with and without the presence of SC (Figure 6A). Notably, the combined use of SC with cisplatin significantly reduced MC38 tumor growth compared to the individual treatments alone (Figures 6B, C). Complementing this finding, photographs of the tumors clearly illustrated the significant retardation of tumor growth in the group treated with combination therapy (Figure 6D), compared to the groups treated individually with either SC or cisplatin. These findings significantly implied a potent combined effect of SC and cisplatin in successfully inhibiting tumor growth *in vivo*.

Exposure to cisplatin was observed to result in significant weight loss in the mice. However, this weight loss was significantly ameliorated in the group receiving combined treatment with cisplatin and SC compared to the cisplatin group (Figure 6E). In addition, the survival rate of mice also improved dramatically with the co-administration of SC and cisplatin, compared to cisplatin-only treatment (Figure 6F). These results highlight the potential of SC in reducing side effects and improving the therapeutic effect of cisplatin.

**FIGURE 7:**
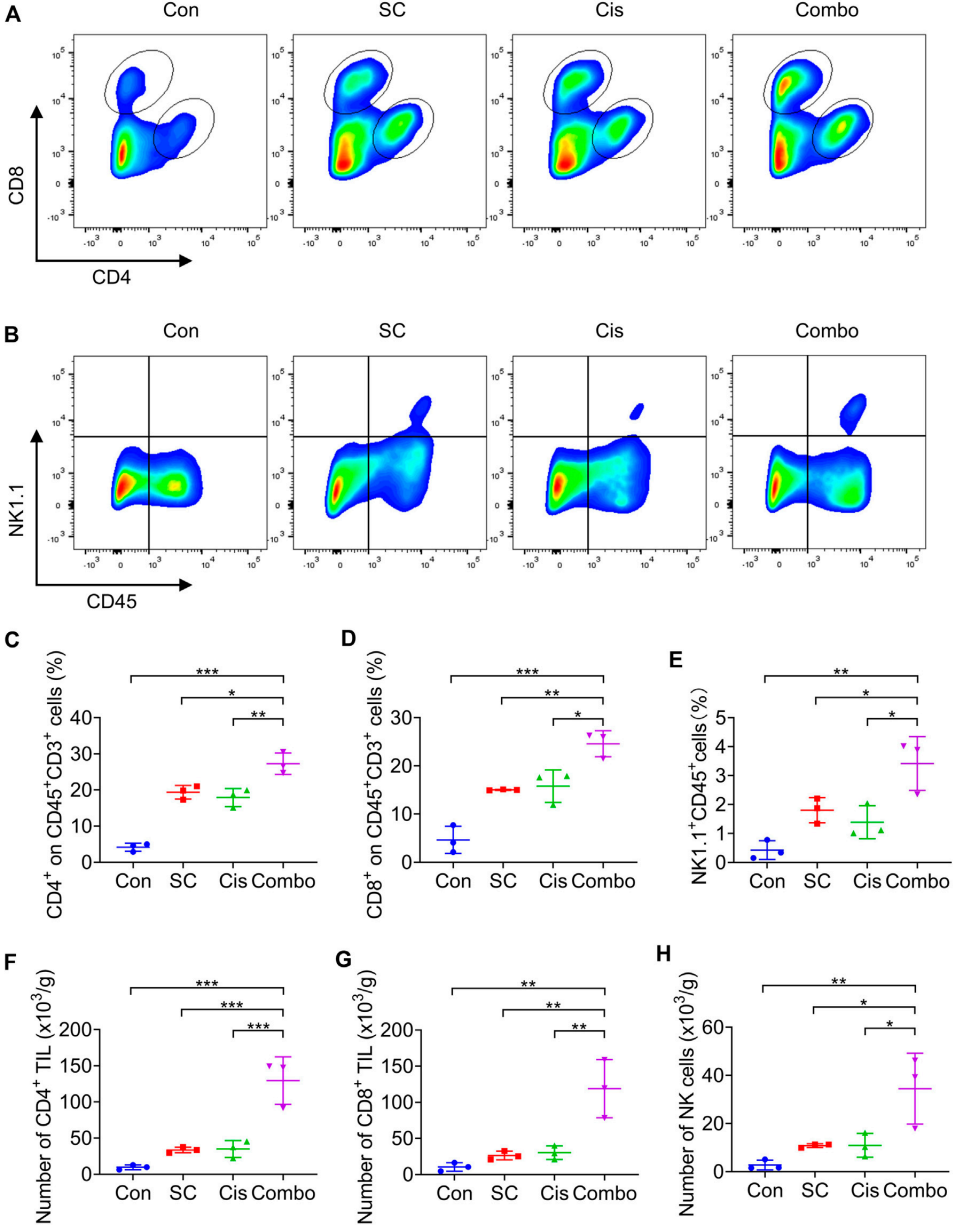
SC enhances the chemotherapeutic effect of cisplatin by boosting T cells and NK cells. **(A,B)** Representative flow cytometry data of CD4^+^ T cells, CD8^+^ T cells, and NK cells in tumors formed by MC38 cells left unstimulated or stimulated with cisplatin (2 mg/kg) in the absence or presence of SC (30 mg/kg). **(C–E)** Quantification of the results from **(A,B)**. **(F–H)** Average numbers of tumor-infiltrating CD4^+^ T cells **(F)**, CD8^+^ T cells **(G)**, and NK cells **(H)** of tissues from transplanted MC38 tumors grown in C57BL/6 mice. Data are shown as mean ± SD, ^*^ *p* < 0.05, ^**^ *p* < 0.01, and ^***^ *p* < 0.001 (one-way ANOVA with Dunnett’s post-hoc test).

Our earlier research illustrated that SC enhances the cisplatin-induced type I IFN response through the cGAS-STING signaling pathway. Accordingly, the mRNA expression of IFNβ, CXCL10, ISG15, and IFIT1 in the tumor tissues was analyzed. The data revealed that SC markedly enhanced cisplatin-induced gene transcription in the tumors established from MC38 cells (Figures 6G–J), underscoring the potentiated effect of SC on the type I IFN response prompted by cisplatin. Flow cytometry analysis confirmed that the combination therapy of SC and cisplatin significantly resulted in an appreciable increase in the infiltration of CD4^+^ and CD8^+^ T cells, along with NK cells, into the tumors (Figures 7A–H). Collectively, SC amplifies the chemotherapeutic efficacy of cisplatin by bolstering antitumor immunity and enhancing the type I IFN response.

## 4 Discussion

SC initially gained attention due to its significant role in reducing serum glutamic-pyruvic transaminase levels and served as a precursor to commonly used hepatitis-lowering drugs such as bifendate and bicyclol in clinical practice (Chang et al., 2009; Szopa et al., 2017; Zhu PL. et al., 2019). Therefore, early research focused on the protective effect of SC on the liver (Jiang et al., 2015), such as improving acetaminophen (APAP)- induced liver injury, anti-liver fibrosis (Dai et al., 2021), and inhibiting HBV replication (Zhao et al., 2023). Beyond this, SC also exhibited outstanding anti-inflammatory (Oh et al., 2010; Park et al., 2013) and antioxidant effects (Kim and Yi, 2018). However, no one has yet focused primarily on its potential antitumor function. For the first time, our work has validated the significant antitumor effect of SC through *in vivo* experiments and connected this activity to the infiltration and activation of CD8^+^ and NK cell caused by the cGAS-STING pathway. Our study has found that SC is a potential antitumor drug. This expands the pharmacological effects of SC and supplements the antitumor immune mechanism of it.

Cisplatin is a widely used chemotherapy drug for the treatment of various forms of cancer and sarcoma (Romani, 2022). The antitumor action of cisplatin is primarily attributed to its ability to cross-link DNA purines, thereby disrupting the DNA repair mechanisms of cancer cells. Finally, it leads to DNA damage and triggers cell apoptosis (Minerva et al., 2023). However, in a significant proportion of cancer patients, the use of cisplatin and its derivatives are hampered by the onset of major side effects, significantly limiting its long-term application (Dasari and Tchounwou, 2014). There remains a pressing need for a combination therapy strategy that mitigates the side effects of cisplatin while enhancing its efficacy. It has been shown that NA from both internal and external sources can activate the cGAS-STING pathway, leading to the activation of functional T cell responses, thereby linking the DNA damage caused by chemotherapy to the stimulation of antitumor immune responses, including the activity of CD8^+^ cytotoxic T cells. Previous research has illustrated that type I IFN can enhance the efficacy of chemotherapy (Salvagno et al., 2019). Consequently, our findings highlight the benefit of using SC alongside platinum-based chemotherapy, increasing the effectiveness of the drug by enhancing antitumor immunity. SC and cisplatin have a synergistic effect in limiting tumor growth *in vivo*, and the use of SC not only enhances the antitumor effect of cisplatin but also reduces its adverse reactions. Intriguingly, during this process, we discovered that the synergistic interaction between SC and cisplatin could bolster the type I IFN response activated by cisplatin. This enhancement relied on the cGAS-STING signaling pathway and encouraged the infiltration of CD4^+^, CD8^+^ T cells, and NK cells into the tumors. Therefore, through our work, we have provided a new option for use in combination with chemotherapy drugs. Identifying compounds that boost cGAS-STING activity could be beneficial for cancer treatment. This approach would leverage STING-mediated antitumor immunity in response to DNA damage caused by chemotherapy in patients.

## 5 Conclusion

This study provides a theoretical basis for the possible use of SC in breast cancer and colon cancer treatment and enriches the antitumor research of it. Moreover, our data confirm that SC can sensitize chemotherapy through antitumor immunity by increasing the cisplatin-activated type I IFN response, which lays the basis for combining SC with platinum-based chemotherapeutics in cancer treatment.

## Data Availability

All relevant data associated with this study have been uploaded to the SRA database and are accessible through the following link: https://www.ncbi.nlm.nih.gov/sra/PRJNA1127210.

## Abbreviations

cGAS, cyclic GMP–AMP synthase; STING, stimulator of interferon genes; IFN, interferon; cGAMP, cyclic GMP-AMP; IRF3, interferon regulatory factor 3; ISGs, interferon-stimulated genes; CTLs, cytotoxic T lymphocytes; SC, Schisandrin C; PCA, Principal Component Analysis; KEGG, Kyoto Encyclopedia of Genes and Genomes; GO, Gene Ontology; GSEA, Gene Set Enrichment Analysis.
